# Plasma osteoprotegerin and breast cancer risk in BRCA1 and BRCA2 mutation carriers

**DOI:** 10.18632/oncotarget.13417

**Published:** 2016-11-22

**Authors:** Lovisa Odén, Mohammad Akbari, Tasnim Zaman, Christian F. Singer, Ping Sun, Steven A. Narod, Leonardo Salmena, Joanne Kotsopoulos

**Affiliations:** ^1^ Women's College Research Institute, Women's College Hospital, Toronto, ON, Canada, M5S 1B2; ^2^ Karolinska Institutet, SE-171 77, Stockholm, Sweden; ^3^ Dalla Lana School of Public Health, University of Toronto, Toronto, ON, Canada, M5T 3M7; ^4^ Department of Obstetrics and Gynecology and Comprehensive Cancer Center, Medical University of Vienna, Spitalgasse 23, 1090 Wien, Vienna, Austria; ^5^ Department of Pharmacology and Toxicology, University of Toronto, 1 King's College Circle Toronto, ON, Canada, M5S 1A8; ^6^ Princess Margaret Cancer Centre, University Health Network, Toronto, ON, Canada, M5G 2M9

**Keywords:** osteoprotegerin (OPG), BRCA1/2, chemoprevention, breast cancer, receptor activator of nuclear factor κB (RANKL)

## Abstract

Emerging evidence suggests a role of receptor activator of nuclear factor κB (RANK)/RANK ligand (RANKL) signaling in breast cancer development. Lower osteoprotegerin (OPG) levels, the endogenous decoy receptor for RANKL which competes with RANK for binding of RANKL, has been reported among *BRCA* mutation carriers. Whether low OPG levels contribute to the high breast cancer risk in this population is unknown. OPG concentrations were measured in plasma of 206 cancer-free *BRCA* mutation carriers using an enzyme-linked immunosorbent assay. Subjects were categorized as high vs. low based on the median of the entire cohort (95 ng/mL) and followed for a new diagnosis of breast cancer. Cumulative incidence by baseline plasma OPG concentration was estimated using Kaplan-Meier survival analysis. Cox proportional hazards models were used to estimate the adjusted hazard ratios for the association between plasma OPG and breast cancer risk. Over a mean follow-up period of 6.5 years (range 0.1–18.8 years), 18 incident breast cancer cases were observed. After ten years of follow-up, the cumulative incidence of breast cancer among women with low OPG was 21%, compared to 9% among women with high OPG (*P*-log rank = 0.046). After multivariate adjustment, women with high plasma OPG had a significantly decreased risk of developing breast cancer, compared to women with low OPG (HR = 0.25; 95%CI 0.08–0.78; *P* = 0.02). These data suggest that low OPG levels are associated with an increased risk of *BRCA*-associated breast cancer. Targeting RANK signalling may represent a plausible, non-surgical prevention option for *BRCA* mutation carriers.

## INTRODUCTION

Women who inherit a deleterious mutation in one of the two breast cancer susceptibility genes, *BRCA1* or *BRCA2*, face a high lifetime risk of breast cancer, compared to women in the general population [1–3]. Genetic testing for mutations in *BRCA1* or *BRCA2* identifies women at a high-risk of developing breast cancer; however, to date the only highly effective primary breast cancer prevention strategy is prophylactic bilateral mastectomy [4–6]. Furthering our understanding of the underlying biology of *BRCA*-associated cancer development should translate into targeted chemoprevention options for this high-risk population.

Evidence from both epidemiologic and experimental studies point towards an important role of progesterone-signaling in breast cancer development. Findings from the Women's Health Initiative (WHI), a randomized placebo-controlled trial of hormone replacement therapy showed that combined therapy (i.e., estrogen plus progesterone) was associated with a significant increased risk of breast cancer (hazard ratio [HR] = 1.55; 95%CI 1.41–1.70) [7] whereas the use of estrogen alone was associated with a decreased risk (HR = 0.77; 95%CI 0.62–0.95) [8]. *In vivo* studies have provided mechanistic evidence to explain these observations. Various groups have demonstrated that progesterone-mediated up-regulation of the receptor activator of nuclear factor κB signaling pathway (RANK)/RANK ligand (RANKL) plays a critical role in mammary gland epithelial cell proliferation, in mammary stem cell expansion, and in carcinogenesis [9–13]. Recently, two independent reports showed that inhibition of RANKL significantly suppressed mammary tumorigenesis in *Brca1* deficient mice [14, 15]. Moreover, Widschwendter *et al*. reported significantly lower mean circulating levels of osteoprotegerin (OPG), the endogenous decoy receptor for RANKL that antagonizes RANK/RANKL-mediated signaling, as well as higher progesterone levels among premenopausal *BRCA* mutation carriers compared to non-carrier controls [16, 17]. Together, these studies strongly suggest that a role for the progesterone-mediated RANK/RANKL signaling pathway in the development of mammary carcinogenesis may be particularly relevant in women with a *BRCA* mutation. Therefore, targeting aberrancies in RANKL signaling may represent a plausible, non-surgical chemoprevention option for this high-risk population.

Despite the emerging importance of RANK signaling in breast cancer, no studies have directly measured the association between circulating OPG levels and subsequent breast cancer risk. Thus, we undertook a prospective analysis of plasma OPG levels and breast cancer risk in 206 women with a *BRCA1* or *BRCA2* mutation that were unaffected by breast cancer at the time of study enrollment.

## RESULTS

There were 206 women with a *BRCA1* or *BRCA2* mutation included in the current study. The baseline characteristics of the women are summarized in Table 1. The median baseline OPG level was 65.2 pg/ml (interquartile range [IQR] 46.7 to 83.3 pg/ml) among women in the low group and 134.9 pg/ml (IQR 112.2 to 191.9 pg/ml) among those in the high group. Women with low plasma OPG levels were younger, on average compared to women with high OPG levels (*P* = 0.008). Other baseline characteristics, including reproductive factors, body mass index, and menopausal status, were similar between the two groups.

**Table 1:** Characteristics of BRCA mutation carriers by plasma osteoprotegerin (OPG) levels

Variable	Low OPG(*n*= 103)	High OPG(*n*= 103)	*P^§^*
Osteoprotegerin (ng/ml), mean (range)	62.9 (4.2–94.5)	168.1 (95.5–547.7)	< 0.0001
Year of birth, mean (range)	1965.0 (1931.4–1990.5)	1959.8 (1911.7–1990.5)	0.008
Age at blood draw, mean (range), years	40.8 (19.6–70.6)	44.2 (17.6–84.7)	0.06
Mean follow-up, years (range)	6.3 (0.2–17.7)	6.6 (0.1–18.8)	0.69
*BRCA* mutation			
*BRCA1*	59 (57.3)	61 (59.2)	
*BRCA2*	44 (42.7)	41 (39.8)	
*BRCA1* and *BRCA2*	0	1 (1.0)	0.57
Oophorectomy, *n* (%)	59 (57.3)	62 (60.9)	0.67
Breast cancer, *n* (%)			
No	90 (87.4)	97 (94.2)	
Yes	13 (12.6)	6 (5.8)	0.09
Age at diagnosis, mean (range)	51.4 (33.9–62.7)	49.1 (35.5–72.4)	0.67
Parity, *n*(%)			
Nulliparous	33 (32.7)	31 (30.1)	
1	9 (8.9)	8 (7.8)	
2	37 (36.6)	34 (33.0)	
3	17 (16.8)	16 (15.5)	
≥ 4	5 (5.0)	14 (13.6)	0.34
Mean (range)	1.5 (0–5)	1.8 (0–7)	0.15
Missing	2	0	
Breastfeeding, *n* (%)			
Never	50 (49.5)	44 (43.1)	
Ever, < 12 months	26 (25.7)	26 (25.5)	
Ever, ≥ 12 months	25 (24.8)	32 (31.4)	0.53
Mean (range), months	8.5 (0–126)	9.6 (0–86)	0.63
Missing	2	1	
Oral contraceptive use, *n* (%)			
Never	26 (25.2)	20 (19.4)	
Ever	76 (73.8)	83 (80.6)	
Missing	1 (1.0)	0	0.35
Body mass index, mean (range)	25.2 (17.2–45.3)	24.6 (17.9–45.1)	0.37
Tamoxifen use, *n* (%)			
Never	102 (99.0)	102 (99.0)	
Ever	1 (1.0)	1 (1.0)	1.00
Menopausal status, *n* (%)			
Premenopausal	77 (74.8)	64 (62.1)	
Postmenopausal	26 (25.2)	39 (37.9)	0.05

* Oophorectomy refers to bilateral oophorectomy.

^§^*P* values were calculated using the Student's *t*-test for continuous variables and the chi-square test for categorical variables. Missing data were not included in the analysis. All tests are two-sided.

**Figure 1:**
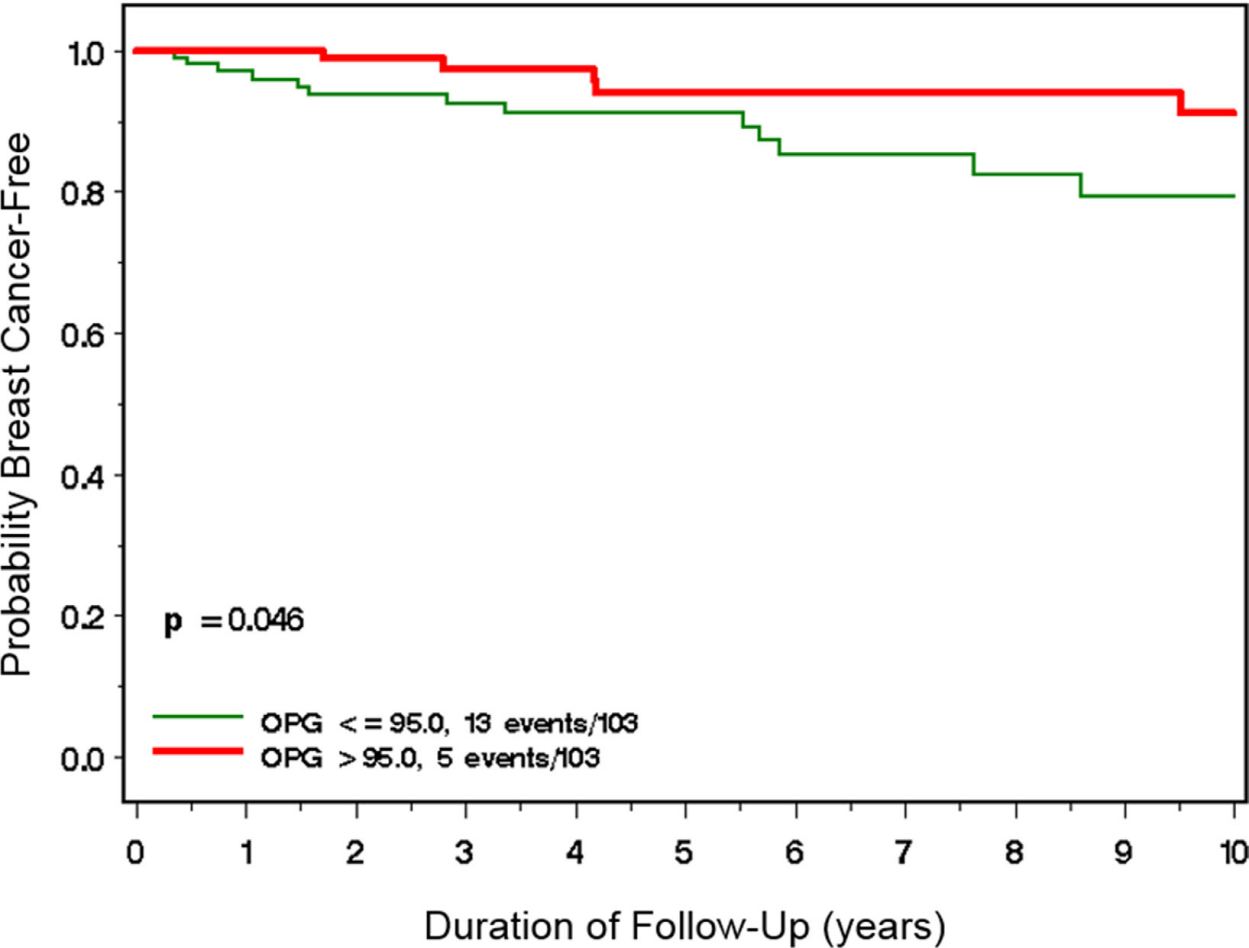
Incidence of breast cancer among BRCA1 and BRCA2 mutation carriers with high (> 95 ng/ml) vs. low (≤ 95 ng/ml) plasma OPG levels

Over a mean follow-up period of 6.5 years (range 0.1–18.8 years), 18 incident cases of primary invasive breast cancer were observed in the cohort. Thirteen (13%) of the women with low OPG developed breast cancer compared to 6 (6%) of the women with high OPG levels (*P* = 0.09). Women who developed breast cancer had significantly lower mean baseline OPG concentrations (90.59 pg/ml [range 4.2–205.7 pg/ml]) compared to the OPG concentrations of women who did not develop breast cancer (117.9 pg/ml [7.4–547.7]) (*P* = 0.04). *BRCA* mutation carriers with low baseline OPG concentrations (≤ 95 pg/ml) had a significantly higher risk of developing breast cancer compared to those with high baseline OPG concentrations (> 95 pg/ml)(Figure 1). After ten years of follow-up, the cumulative incidence of breast cancer among women with low OPG concentrations was 21% (95%CI 12%–35%) compared to 9% (95%CI 4%–21%) among women with high OPG concentrations (*P*-log rank test = 0.046).

Table 2 summarizes the hazard ratio (HR) and associated 95% confidence intervals (CI) of breast cancer associated with plasma OPG levels among *BRCA1* and *BRCA2* mutation carriers. After adjusting for age at blood draw, women with high plasma OPG levels had a significantly decreased risk of developing breast cancer compared to women with low plasma OPG levels (HR = 0.30; 95% CI 0.10–0.86; *P* = 0.02). Results were similar in the multivariate analysis adjusting for potential confounders including *BRCA* mutation type, oophorectomy, breastfeeding and oral contraceptive use (HR = 0.25; 95% CI 0.08–0.78; *P* = 0.02)(Table 2). In the analysis stratified by *BRCA* mutation, the adjusted estimates were 0.39 (95% CI 0.08–1.85; *P* = 0.24) for *BRCA1* and 0.10 (95% CI 0.01–0.95; *P* = 0.04) for *BRCA2* mutation carriers (data not shown).

**Table 2:** Hazard ratio (HR) and 95% confidence interval (CI) of breast cancer by plasma osteoprotegerin (OPG) levels

Variable	Age-adjusted*HR (95% CI)	*P*-value	Multivariate§HR (95% CI)	*P*-value
Plasma osteoprotegerin (OPG)				
Low	1.00 (reference)	0.02	1.00 (reference)	
High	0.30 (0.10–0.86)		0.25 (0.08–0.78)	0.02
Age at blood draw (continuous)	1.04 (1.00–1.08)	0.04	1.05 (1.01–1.10)	0.02
*BRCA* mutation type				
*BRCA1*	1.00 (reference)	0.80	1.00 (reference)	
*BRCA2*	1.11 (0.44–2.80)		0.93 (0.35–2.51)	0.89
Oophorectomy				
No	1.00 (reference)		1.00 (reference)	
Yes	0.49 (0.19–1.27)	0.14	0.34 (0.11–1.04)	0.06
Breastfeeding				
Never	1.00 (reference)		1.00 (reference)	
<1year	1.50 (0.51–4.43)	0.46	1.89 (0.60–5.97)	0.28
≥1year	0.81 (0.24–2.71)	0.76	1.03 (0.30–3.57)	0.96
Oral contraceptive use				
Never	1.00 (reference)		1.00 (reference)	
Ever	1.15 (0.35–3.73)	0.82	2.17 (0.55–8.54)	0.27

* Adjusted for age at blood draw.

^§^ Adjusted for age at blood draw as well as other variables in the model (*BRCA* mutation, oophorectomy, breastfeeding and oral contraceptive use).

## DISCUSSION

In this study, we demonstrate a significant inverse relationship between plasma OPG levels and breast cancer risk among 206 women with an inherited *BRCA1* or *BRCA2* mutation. Women with low OPG levels were more likely to develop breast cancer compared to women with high levels. These findings are timely given the emerging importance of RANK/RANKL-signaling in mammary tumour development, which may be of more relevance for *BRCA* mutation carriers [14, 15]. Normally, OPG regulates RANK/RANKL-signaling by binding to RANKL as a decoy receptor and thereby inhibiting signal transduction [18]. Recent studies have described progesterone-mediated RANKL signaling on ER/PR-negative epithelial stem and progenitor cell populations as a mechanism contributing to mammary tumorigenesis [19, 20]; however, the direct role of OPG on risk of developing *BRCA*-breast cancer has not been evaluated. Our data adds to a rapidly emerging body of evidence that a *BRCA* mutation may be associated with aberrancies in the OPG/RANK- signaling axis and may represent a potential therapeutic target for the non-surgical prevention of breast cancer in this high-risk population.

Our results extend the findings of Widschwendter *et al*., who demonstrated lower serum OPG levels among *BRCA* mutation carriers (*n* = 391) compared to non- carriers (*n* = 782) across the menstrual cycle (*P* = 0.06) [17]. They also reported that progesterone levels were higher during day 6 to day 24 (i.e., luteal phase) of the menstrual cycle of *BRCA* mutation carriers (particularly *BRCA1* mutation carriers) vs. non-carriers. Of importance was a significant inverse relationship between luteal progesterone and OPG among women with a mutation (ρ = −0.216; *P* = 0.002) vs. those without (ρ = −0.098; *P* = 0.06). Using female cynomolgus macaques, the authors showed that treatment with estrogen plus progesterone (but not estrogen alone or no hormones) was associated with significantly lower mammary tissue and serum OPG levels. Importantly, there was a significant inverse correlation between serum OPG levels and mammary gland proliferation as per Ki67 expression analysis (rho = −0.545; *P* < 0.001). Although estrogen plus progesterone was also associated with an upregulation of RANKL in the mammary tissue, this was not reflected in the serum. These findings suggest a local effect of progesterone that could explain the tissue-specificity of cancer development in response to aberrant OPG/RANKL levels.

To our knowledge, our study represents the first investigation of plasma OPG levels and breast cancer risk in *BRCA* mutation carriers. Our study is consistent with a recent and only evaluation of serum OPG levels and subsequent cancer risk in non-carriers. Among 6,279 subjects from the general population in Norway with a median follow-up of 13.5 years (3,174 women), Vik *et al*., reported a significant inverse relationship between serum OPG levels and breast cancer risk (HR tertile 3 vs. tertile 1 = 0.24; 95%CI 0.10–0.61; *P* – trend = 0.002) [21]. Although the expected number of mutation carriers is likely very small in this study, it is of interest that the protective effect of high circulating OPG was only seen in premenopausal women, given that *BRCA* mutation carriers typically present disease at a young age (< 50 years) [22]. In our study, the average age at diagnosis was 50.7 and 37% were diagnosed prior to age 50.

Evidence from experimental studies strongly support a role of progesterone-mediated RANKL signaling in the development of *BRCA*-associated breast cancer. *In vivo* studies have demonstrated that progesterone is directly (via binding to the progesterone receptor [PR]) and indirectly (via RANKL) involved in normal mammary gland development as well as mammary tumorigenesis [10, 11, 23]. Specifically, binding of RANKL to RANK in response to progesterone stimulates mammary epithelial cells maturation and proliferation. Inhibition of either RANKL or progesterone by pharmacological agents or inactivation of RANK are associated with a reduction in mammary carcinogenesis [10, 11, 23]. Conversely, overexpressing RANK enhances mammary tumor formation in the presence of progesterone [11] while *in vivo* administration of a synthetic progesterone upregulates RANKL levels in mammary epithelial cells [10]. Poole *et al*., demonstrated that treatment of *brca1/p53*-deficient mice with the progesterone antagonist mifepristone prevented mammary tumorigenesis [23]. Epidemiologic evidence also supports a harmful role of progesterone, given that only combined hormone replacement therapy (i.e., estrogen plus progesterone) has been associated with an increased risk of developing breast cancer [24].

Of particular importance are studies demonstrating that progesterone-driven RANKL-signalling is implicated in the regulation and expansion of mammary stem and progenitor cells [11–13]. Joshi and colleagues have showed progesterone-mediated expansion of the luminal progenitor cells in human breast tissue which is of direct relevance for *BRCA1* mutation carriers who have an increased propensity to develop breast tumours with stem cell-like properties [25]. Specifically, emerging evidence points to the luminal progenitor population as the cell of origin of *BRCA1*-associated breast tumours [26]. Accordingly, Lim *et al*., showed that breast cells isolated from *BRCA1* mutation carriers exhibit an expanded luminal progenitor subpopulation compared to normal mammary tissue [26]. In a recent publication, Nolan *et al*., demonstrated that RANK expression was highest in the luminal progenitor cells (normal breast and tumour tissue) of *BRCA1* mutation carriers compared to tissue from both wild-type and *BRCA2* mutation carriers [14]. They also showed a decrease in breast epithelial cell proliferation in three premenopausal women treated with denosumab, and a significant increase in basal-like tumor latency in mammary hyperplasia among *Brca1* deficient mice (that also develop) treated with a RANKL inhibitor (15). Importantly, these findings were validated in a concomitant study led by Sigl and colleagues [15].

The annual risk for breast cancer in *BRCA* mutation carriers is ∼2% between the ages of 30 and 60. Ideally, a drug intervention would need to occur early in adulthood and have no serious or long-term side effects. Quantifying OPG levels could help stratify women who are at a higher risk of developing disease who may be targets for alternative chemoprevention options. The low endogenous levels of OPG coupled with the significant lifetime risks of breast and ovarian cancer in *BRCA* mutation carriers, strongly support the use of an anti-RANKL monoclonal antibody like denosumab for the prevention of hereditary breast cancer while simultaneously maintaining bone density. Last year, authors of the adjuvant denosumab (a monoclonal antibody that binds RANKL) in breast cancer trial (ABCSG-18) published no additional toxicity and a favorable safety profile of adjuvant denosumab therapy (60 mg subcutaneously biannually) among women with early breast cancer treated with aromatase inhibitors compared to the placebo group [27]. Furthermore, a recent follow-up analysis of the trial showed improved disease-free survival among the women receiving adjuvant denosumab [28]. An effect on the incidence of a second primary cancer will be of interest.

Our study has several limitations. The sample size was small, the number of incident cases was low and the finding was of marginal statistical significance. We were not sufficiently powered to conduct robust analyses subdivided by *BRCA* mutation type. Although we did not have data on timing of blood collection, Widschwendter *et al*. clearly showed no variation of OPG within the menstrual cycle and there was no difference in OPG levels by menopausal status in our cohort of *BRCA* mutation carriers (data not shown). We did not evaluate levels of RANKL in our analysis. Despite this, the recent findings from Nolan and colleagues of increased RANK in *BRCA1* luminal progenitors suggests that it may be of interest to evaluate circulating RANKL levels in future studies with a much larger number of women.

Notwithstanding these limitations, this is the first report of circulating OPG and breast cancer risk among women with a *BRCA* mutation. Pending validation in a larger study, the integration of OPG levels may help identify those women who may benefit from treatment with RANKL blockade to prevent breast cancer. Inhibiting RANK-signaling by an existing monoclonal antibody (i.e., denosumab) to simultaneously prevent breast cancer and maintain bone health (particularly after salpingo-oophorectomy) represents an important opportunity that warrants exploration in a primary prevention trial of *BRCA* mutation carriers.

## MATERIALS AND METHODS

### Study population

We identified potentially eligible women from a longitudinal study of *BRCA1* and *BRCA2* mutation carriers [29]. For the current prospective cohort study, we included women from Ontario, Canada, who had received genetic counselling at the Familial Breast Cancer Research Unit, Women's College Hospital (Toronto, Canada). These women sought testing for *BRCA1* or *BRCA2* mutations because of a personal or family history of breast and/or ovarian cancer. Mutation detection was conducted using a range of techniques, but all nucleotide sequences were confirmed by direct sequencing of DNA. The study was approved by the institutional ethics review boards of the host institutions and all study subjects provided written informed consent.

### Data and sample collection

All study subjects completed a baseline questionnaire at the time of study enrolment. These were either mailed to each study participant or administered over the phone by a genetic counsellor or research assistant at the time of genetic testing and collected information regarding family history, reproductive and medical histories, preventive oophorectomy and mastectomy, and select lifestyle factors such as alcohol and coffee consumption. Follow-up questionnaires were completed every two years thereafter to update information on relevant reproductive, medical and lifestyle information, and to ascertain incident cancers.

Blood samples were collected at the time of genetic testing and baseline questionnaire administration into EDTA- or citrate-containing tubes by venipuncture, processed and separated into plasma and DNA and stored at −80°C and 4°C respectively, at the Women's College Hospital.

### Study subjects available for analysis

Women were eligible for inclusion in the current study if they were between the ages of 18–70 years at the time of enrolment and completed at least one follow-up questionnaire. Of the 751 women who were initially eligible, we excluded those who had a previous history of breast or ovarian cancer (*n* = 284), those who had previously undergone a prophylactic bilateral mastectomy (*n* = 11), and those who did not have a baseline plasma sample available (*n* = 250). After these exclusions, a total of 206 participants were available for the plasma OPG analysis.

### OPG ELISA

Plasma OPG was quantified using a commercial ELISA kit from ALPCO (Salem, NH – catalogue #04-BI-20403) according to the manufacturer's protocol. Briefly, all patient plasma samples were run in duplicate. The concentration of OPG was calculated as the average of duplicate samples (each adjusted for background signal and normalized to blank wells) and subsequently converted to a total OPG concentration upon comparison to OPG standards provided in the kit.

### Statistical analysis

Plasma OPG was categorized as high or low based on the median distribution in the entire cohort (≤ 95 and > 95 ng/mL). The student's *t-test* and χ^2^ test were used to evaluate differences in the baseline characteristics of the women with high vs. low OPG levels.

Participants were followed from date of completion of the baseline questionnaire until either the date of their breast cancer diagnosis, date of prophylactic mastectomy, date of ovarian cancer diagnosis, date of death or date of completion of their last follow-up questionnaire. The follow-up period of this analysis was from the date of baseline questionnaire until June 26^th^, 2016.

We used the Kaplan-Meier survival analysis to estimate the cumulative incidence of breast cancer in women with high vs. low plasma OPG levels. Incidence rates in the two groups were compared using a log-rank test. We employed Cox proportional hazards models to estimate the hazard ratio (HR) and 95% confidence interval (CI) associated with plasma OPG levels. The multivariable model was adjusted for age at blood draw (continuous), *BRCA* mutation type (*BRCA1* or *BRCA2*), oophorectomy (yes/no), breastfeeding (never, < 1 year, ≥ 1 year) and oral contraceptive use (never, ever). All analyses were conducted using SAS version 9.4 software (SAS Institute, Cary, NC). All *P* values were 2-sided and were considered statistically significant if *P* ≤ 0.05.
